# Miscellaneous Items

**Published:** 1890-02

**Authors:** 


WHAT IS IT THAT PULLS A PERSON DOWN?—It is not intellectual work that injures the brain, says the London *Hospital*, but emotional excitement. Most men can stand the severest thought and study of which their brains are capable and be none the worse for it, for neither thought nor study interferes with the recuperative influence of sleep. It is ambition, anxiety and disappointment, the hopes and fears, the loves and hates of our lives that wear out our nervous system and endangers the balance of the brain.

"QUACKERY feeds upon the ignorance and credulity of the general public, and yet, newspapers, the promoters of intelligence and the bulwarks of morals, have no word for it but of praise, if it will only advertise, while the 'regulars' who do so much for the poor and unfortunate are berated in a style that self rspect forbids any notice of."—*St. Joseph Med. Herald.*

AMMONIA IN THE BATH.—Nothing so quickly restores tone to exhausted nerves and strength to a weary body as a bath containing an ounce of aqua ammonia to each pail of water. It makes the flesh firm, and smooth as marble, and renders the body pure and free from odor.—*Annals of Hygiene.*

IN Germany the government has come to the conclusion that there are enough medical colleges in the country and refuses to allow any more to be organized.

MASCULINE SUPERIORITY.—"I see that a post mortem examination is often made in murder cases. What does a post mortem examination mean?" asked a young woman of her better half.

"A post mortem examination, my dear, is intended to allow the victim to state, verbally, his own testimony against his assailant, and is taken down in writing."

"Thanks, darling; and you won't look down on me, will you, because I haven't your education?" He said he wouldn't.—*Med. and Surg. Reporter.*

